# Training or Synergizing? Complex Systems Principles Change the Understanding of Sport Processes

**DOI:** 10.1186/s40798-020-00256-9

**Published:** 2020-07-13

**Authors:** Rafel Pol, Natàlia Balagué, Angel Ric, Carlota Torrents, John Kiely, Robert Hristovski

**Affiliations:** 1 Real Federación Española de Fútbol (Spain), Complex Systems in Sport Research Group, Institut Nacional d’Educació Física de Catalunya (INEFC), University of Lleida (UdL), Complex de la Caparrella, s/n, 25192 Lleida, Spain; 2 Complex Systems in Sport Research Group, Institut Nacional d’Educació Física de Catalunya (INEFC), University of Barcelona (UB), Av. de l’Estadi, 12-22, 08038 Barcelona, Spain; 3 FC Barcelona, Barcelona (Spain), Complex Systems in Sport Research Group, Institut Nacional d’Educació Física de Catalunya (INEFC), University of Lleida (UdL), Complex de la Caparrella, s/n, 25192 Lleida, Spain; 4 Complex Systems in Sport Research Group, Institut Nacional d’Educació Física de Catalunya (INEFC), University of Lleida (UdL), Complex de la Caparrella, s/n, 25192 Lleida, Spain; 5 Institute of Coaching and Performance, School of Sport and Wellbeing, University of Central Lancashire, Preston, PR1 2HE UK; 6 Complex Systems in Sport Research Group, Faculty of Physical Education, Sport and Health, Ss. Cyril and Methodius University, Dimche Mirchev, 1000, Skopje, North Macedonia

**Keywords:** Team synergies, Nonlinear dynamics, Nested organization, Timescales, Diversity potential, Constraints

## Abstract

There is a need to update scientific assumptions in sport to promote the critical thinking of scientists, coaches, and practitioners and improve their methodological decisions. On the basis of complex systems science and theories of biological evolution, a systematization and update of theoretical and methodological principles to transform the understanding of sports training is provided. The classical focus on learning/acquiring skills and fitness is replaced by the aim of increasing the diversity/unpredictability potential of teams/athletes through the development of synergies. This development is underpinned by the properties of hierarchical organization and circular causality of constraints, that is, the nestedness of constraints acting at different levels and timescales. These properties, that integrate bottom-up and top-down all dimensions and levels of performance (from social to genetic), apply to all types of sport, ages, or levels of expertise and can be transferred to other fields (e.g., education, health, management). The team as the main training unit of intervention, the dynamic concept of task representativeness, and the co-adaptive and synergic role of the agents are some few practical consequences of moving from training to synergizing.

## Key Points

- The fittest are not necessarily the strongest or fastest but the most diverse.
- Diversity is developed by creating synergies through the strategic manipulation of constraints.
- The interdependence, temporal nestedness, and circular causality of constraints acting at different levels and timescales integrate all dimensions and levels of performance in a correlated way
- Synergizing, instead of training, defines an improved understanding of the process and helps scientists, coaches, and practitioners to create safer and effective interventions.

## Introduction

In recent decades, sports training has rapidly evolved, in large part, as a consequence of science-led advances [1]. However, some core assumptions and methodologies have remained unchallenged and unchanged despite the fact that their underpinning theories have disintegrated [2]. Recent research suggests that coaches commonly acquire coaching knowledge from informal, self-directed learning sources and subsequently approach new information in an inefficient fashion [3, 4], thereby limiting practitioners development of open-mindedness, self-reflection, and critical thinking skills. In fact, when expert coaches’ perceptions and practices are studied, attention is most commonly placed on *what* they do, rather than *why* and *how* they do it [3, 5]. The subsequent presumption that practical experience is more relevant than scientific theories, accordingly, is commonplace within coaching cultures and may in part explain the prevalence of pseudoscience in professional practice [6]. The methodologies inspired by successful practitioners are often perpetuated, yet rarely questioned. Academic insights, in contrast, are frequently ignored and discounted. Sports and exercise physiology and psychology, perhaps the two most influential sports science disciplines, are characterized by a strongly reductionist philosophy and remain largely impervious to the transdisciplinary and holistic theories emanating from the science of complex systems [4, 6]. This explains why reductionist thinking persists across the sports sciences—even in recently emerging integrative approaches focused on skill acquisition and interpersonal coordination [7, 8]. Eccentric strength and lactate-based endurance training programs are two well-known examples of reductionist approaches to sport training. Eccentric exercise programs are based on the assumption that high muscle strains, resulting from large tensile forces, drive more advantageous tissue re-modelling as shown when testing single fibers in vitro [9]. This assumption, however, ignores that the critical tensile forces that produce strain in vitro cannot be applied to muscles in vivo [9]. Nonetheless, despite their low level of evidence and potential adverse effects [10–12], eccentric training programs are extensively promoted to prevent injuries [13]. Similarly, lactate-based training prescriptions, which equate blood lactate concentrations to internal load, are commonplace in endurance sports [14]. In this context, the monitoring and modulation of heart rates, corresponding to specific blood lactate concentrations, are used to regulate external loads. Clearly, however, there is a significant uncoupling between internal and external loads under the acknowledged influence of multiple ever-varying contextual interactions [15]. Similarly, the segregation of performance into distinct dimensions (physical, technical, tactical, cognitive) and the consideration of players/dyads as the main training units in team sports, instead of the whole team, are other examples of the strong reductionist influence permeating sports science domains.

Clearly, there is a need to update theoretical training assumptions on the basis of advances in neuroscience and dynamic complex systems science. However, potential training innovations frequently encounter a resistance to change within coaching contexts [2, 5]. For instance, one may ask: (a) are complex science based methodologies really new? Or (b) if traditional training methodologies are tried and tested [16], why should they be changed? Why adopt methodologies of unknown efficacy? After all, increasing practice variability, during interventions like small-sided and conditioning games, was already a feature of sports practice before complex systems methodologies were developed.

What is relevant to emphasize here, however, is that such interventions were typically sporadically used and for the most part were substantiated only on the basis of experiential and intuitive knowledge, without clear insight or academic rationalization as to *why* such interventions offer enhanced outcomes. Importantly, understanding enables generalization and functional transfer of the application to different contexts. In fact, training interventions are not intrinsically valid or invalid but contextually more (in)appropriate or (un)functional. For instance, a strict prescription can be adequate for a stressed novice (e.g., before a penalty kick) but inadequate for an expert player. Recognizing the appropriateness of certain interventions demands that sport scientists and coaches do not simply rely on personal, and inevitably biased, interpretations of their own experiences. Effective training judgement and decision-making require a deep understanding of the properties of the systems (athletes/teams), the principles that dictate their interactions within the environment, and well-defined process objectives. Here, we propose to improve the understanding of such properties and principles on the basis of complex systems and evolutionary biology. Under the complex systems framework, we include numerous theoretical and practical approaches of different spectrum (more general and more applied) sharing principles: synergetics, nonlinear science, dynamic systems, coordination dynamics, ecological physics, ecological dynamics, nonlinear pedagogy, differential learning, etc. To avoid confusion, we are not suggesting a new term to describe this contribution. It is not a new method or approach, it is simply an attempt to question old assumptions and in so doing to improve the understanding of sport scientists, coaches, and practitioners. The final aim is to promote safer and more efficient interventions across all sports, ages, and levels of expertise. The basis of these assumptions is rooted in referenced works, mainly drawing on the evidence base underpinning the constraints-led approach (CLA). The hypothesized, and novel, principles subsequently provide a background for future experimental research.

First, we propose a systematization and extended application of theoretical and methodological principles, based on dynamic complex systems [17, 18], biological evolution [19], and the CLA [1, 17, 18]. Secondly, we emphasize underexplored aspects of the CLA, such as, the interdependent and temporally nested organization of constraints, and the classification and focus of task constraints. Finally, we highlight the safety aspects of the contribution to promote the integration of prevention and performance training. Tables 1 and 2 summarize and contrast the theoretical and methodological principles of the process defined as training^1^ (traditional approach) and synergizing^2^ (complex systems-based approach), respectively, developed in the text.

**Table 1:** Training or synergizing? Contrast of theoretical principles

Approach	Training (traditional)	Synergizing (complex systems)
Conception of athletes/teams	Machines	Complex adaptive systems
Conception of sport	Static entity	Dynamic entity
Scientific approach	Cybernetic Control Theory	Dynamic Systems Theory
Relations among components	Linear cause-effect	Nonlinear dynamic interactions
Integrating mechanisms	Control loops	Circular causality
Control	Internal/external programs	Spontaneous synergies
Organization	Externally designed	Self-organized
Adaptive properties	Homeostasis	Homeodynamics, synergetic reorganization, degeneracy, pleiotropy
Training goal	Maximizing performance attributes	Satisficing diversity/unpredictability potential
Training periodization	Pre-programmed	Co-adapted

**Table 2:** Training or synergizing? Contrast of methodological principles

Approach	Training (traditional)	Synergizing (complex systems)
Programs	Fixed training programs	Contextually sensitive methodological criteria
Performers	Executers	Co-designers of the process
Periodization	Fixed, decontextualized	Contextually sensitive
Conditioning, skill acquisition, motor abilities training	Prescription-based	Based on nested dependence and circular causality of constraints
Training unit	Performers and their components Players (team sports)	Performer-environment system Team (team sports)
Short-term training plan	Based on stereotyped performance solutions and movement templates	Based on exploration of representative performance contexts
Training tasks	Non-representative (through task decomposition)	High level of representativeness (through task simplification) and beyond
Training exercises criteria	Right/wrong	Contextually (un)functional
Evaluation	Fragmented	Holistic
Role of the coach	Prescribing solutions	Co-discovering with the performer

## Training or Synergizing? Contrast of Theoretical Principles

### Conception of Athletes/Teams. Machines or Complex Adaptive Systems?

Individual athletes, teams, and sport games have recently been viewed as complex adaptive systems (CAS) [20–23] whose behavior evolves in response to physical and informational constraints^3^ (e.g., opponent’s actions) [25]. From this perspective, athletes and teams are conceptualized as dynamic complex systems interacting non-linearly, i.e., co-adaptively, with the environment. This perspective contrasts with the conceptualization of human organisms as closed systems (e.g., machines or technical devices) with clearly separable cause-effect relations among components, time-invariant functions, and regulation profiles [26]. Under the framework of dynamic complex systems theory, the behavior of CAS cannot be understood independently from its context, and the training unit is the performer-environment system [27].

Due to the multilayer dynamics of environmental and personal constraints evolving and interacting at different time scales [28], sport is a dynamic entity which itself evolves with the transformation of performers, coaches, equipment, facilities, rules, etc. All these dynamically interacting and co-modulating factors change the pretended prototypic attributes of each sport (e.g., conditional requisites, skills, tactics).

### Integrating Mechanisms. Control Loops or Circular Causality? Pre-programmed Processes or Spontaneous Synergies?

A key property of CAS is the spontaneous formation of structural and functional couplings among components (synergies) to achieve task goals [27, 29, 30]. During sport practice, many degrees of freedom operating at diverse scales (from cellular to social) are continuously re-organized, forming functional goal-oriented synergies, i.e., coordinative structures that allow the reciprocal compensation of components. These synergies, defined at many levels (e.g., muscular, physiological, psychobiological, see [27, 29, 31–34]), constitute embedded coalitions of molecules, muscles, neurons, etc. In the context of the performer-environment system, they tend to operate as unitary ensembles constrained by opponent’s actions or achievement challenges [20]. As each level is nested in the next one, functions are dynamically coupled, and there is no need of a template or plan to rule the relations. In the context of team sports, this entails that, from cellular processes (e.g., biochemical) to collective team synergies (tactical behavior), all functions are dynamically integrated without the need of internal or external programs.

When imposing constraints (variability) on the system, the coupled components in the synergy change together, rather than independently. Thus, instead of the pre-programmed circuits and feedback loops that control and integrate machine functionality, in CAS synergies emerge spontaneously and have circular causal relations with components: thus, components form synergies and those synergies, in turn, govern the components’ behavior [35].

Traditional training approaches, focused on training components (e.g., players in team sports, aerobic and anaerobic metabolic pathways in physical conditioning), ignore that those components are coupled and have integrating properties that feedback, and feedforward circuits do not have. The self-assembled, adaptive interactions drive structural and functional variability, and underpin robustness-enabling properties of CAS such as degeneracy (structurally different components can produce the same function) and pleiotropy (the same components may be assembled to produce multiple functions) [35, 36]. Such properties enable the capacity of CAS to switch between diverse coordinative states while maintaining metastable dynamics [37].

### Training Goal. Maximizing Performance Attributes or Satisficing Diversity/Unpredictability Potential?

In the context of biological evolution, when synergies prove to be functionally advantageous, synergistic selection and stabilization occurs. To maintain fitness and survive within competitive environments, athletes and teams must have sufficient in-group predictability (among support teams and teammates) to maintain coherent behaviors, yet, must be sufficiently unpredictable to disrupt opponents' strategies [38]. Here, the term *teammates* is understood in a wide sense (includes staff managers, etc.) and thus, is also valid for individual sports. Cooperation and competition, the two pillars of biological evolution that rule living systems behavior [19], are basic principles in sports. Contrary to common assumption, the fittest^4^ are not necessarily the strongest, nor the fastest, but the most diverse. Developing strength or velocity is just a means to gain diversity potential [38]. Particularly in sports like football, where the stability and reproducibility of game situations is rare, teams/players continuously deal with a highly unstable non-cooperative environment. In such contexts, survival (in the tournament, championship or league) is defined by positive competition results, which are better achieved through a higher diversity potential.

Because CAS competitors co-adapt, the dynamic stability of survival over long timescales can only be achieved through a continuous process of complexification, i.e., diversification and specialization of performance [19]. This is also true for other sports like gymnastics, athletics, or cyclic sports, where the environment is much more predictable. For instance, a gymnast has more chances to become dynamically stable (i.e., more competitive) by specializing and diversifying the elements of his/her floor routine. This process of complexification is defined by the athlete/team diversity/unpredictability potential [38]. This potential subsumes, but it is not equal to, the diverse functional synergies (reciprocal compensations) coping with diverse unpredictable environments created by the opponent’s behavior and/or challenging environments (e.g., the height of a pole vault). These properties may, or may not, be based on degeneracy. Degeneracy refers to the capacity of attaining similar outcomes with structurally different components. However, diversity and unpredictability also include decision-making processes, e.g., the change of an intended outcome or the space of outcomes altogether. For example, unpredictability can come from a player making a pass (that is, outcome) using structurally different components (that is, different neuro-musculo-skeletal components). However, a player may simply change the desired outcome (shooting or stopping instead of passing). Certainly, this may arise from changes in readiness to act on certain affordances. However, in this case, unpredictability or diversity does not come from degeneracy. Also, a player can change the intended outcome but continue to use the same motor pattern to attain the newly intended outcome. For instance, a player runs to intercept the ball (the initially intended outcome), then decides to let the ball pass since the teammate attains a better position for scoring (changes the intended outcome), but continues to run (maintains the same motor pattern) to mark a defender that tackles his teammate (third intended outcome). These and similar cases do not reduce action unpredictability and diversity to degeneracy alone. On another level of argument, it is important to emphasize that unpredictability is also a relational variable that arises within the performer-environment system. For unpredictability to exist, there must be an opponent striving to anticipate. Without the opponent, the athlete may be diverse, but not unpredictable. Variability, diversity, or degeneracy, on the other hand, refer to properties of an organism or a team alone**.**

It is important here to clarify the term *potential*. This term is used to signify that individuals and teams do not always have to exhibit high diversity of actions, if not constrained to do so. They only exhibit such high diversity of actions if the environment requires it [39–43]. Accordingly, the diversity potential of actions is distributed within the performer/team-environment system, and as such, represents a systemic property. Based on the relation of sport performance in non-cooperative environments with the diversity/unpredictability potential of athletes/teams [38], we emphasize that effective interventions should focus on:

a) *Increasing the athlete/team unpredictability potential* through the formation of new synergies at all levels. By forming new potential synergies, and becoming sensitive to each other’s affordances, i.e., increasing the organization (predictability) of performers within teams, they become more unpredictable for the environment (opponents). The emergence of coordinated behaviors in sports teams is based on the formation of interpersonal synergies between players resulting from collective actions predicated on shared affordances [44]. Higher coordination means dimension reduction and mutual compensation [35, 45], due to the higher co-variation, or mutual information, between the players. Higher co-variation or mutual information means less within team (internal) unpredictability and, consequently, by definition, higher predictability (certainty) due to the mathematical meaning of these measures [35, 46]. This is why sometimes teammates train elaborate schemes of actions and passes, in order to create predictable within-team patterns of activity, which will be not so predictable to opponents. This is termed *functional diversity/unpredictability*. Accordingly, on average, players’ behaviors are coordinated and more predictable within the team, than to the opponents. From the perspective of the athlete/team, the environment becomes more predictable. From the perspective of the opponents (environment), the athlete/team becomes more unpredictable. Athletes/teams who have more diverse degenerate options are more unpredictable and, accordingly, have greater competitive/performance potential.

b) *Have a sufficing diversity/unpredictability potential with respect to the opponent.* Co-adaptivity between opponents is driven by the principle of *sufficing* [47, 48]. This means that opposing athletes/teams always seek, not necessarily to maximize diversity/unpredictability (reaching the global optimum which is typically unattainable), but to develop a sufficiently large potential relative to their opponents (environment), thereby increasing their chances of winning. Performers seek context dependent local optima, i.e., best solutions under local context, that is, local set of constraints.

(c) *Non-decreasing unpredictability potential when constrained by the environment.* Robustly degenerate athletes/teams recover fast by increasing the unpredictability potential, when challenged by the environment and/or the opponent’s actions. The level to which they recover unpredictability is regulated by the second (satisficing, a portmanteau combination of the words satisfy and suffice) principle. Athletes/teams do not use all their diversity/unpredictability potential during all competitions. Instead, they suffice to an appropriate level. This sufficing potential enables a level of diversity/unpredictability that promotes survivability and increases the probability of winning.

The larger degree of diversity potential is individual and realized through diverse actions, which may be defined at different levels and scales (e.g., neuromuscular, cardio-respiratory, (multi)joint, emotional, inter-personal).^5^ Due to synergies, one attribute can be compensated through the development of others to satisfy the task goal. Accordingly, the aim of a synergizing process is not to maximize performance attributes/dimensions but to develop satisficing diversity potential. This means learning to detect the sufficing threshold promoting survival and/or winning. Detecting the level to which the diversity/unpredictability potential has to be unleashed, depending on the opponent, is of utmost importance to athletes’ and teams’ performance success. High performance teams usually lose against less able teams because of this misdetection of sufficing diversity potential engagement. Specific training methodologies may be needed to develop this specific ability. Due to the degenerate properties of CAS [36], there are always alternate ways to achieve the task goal constraint. Hence, while some athletes/teams may gain diversity through the development of physical conditioning, others can do it through the development of coordinative motor skills. For instance, in achievement sports like 100 m running some sprinters can perform dominantly developing a high muscle power and others through more refined coordination patterns. Thus, it does not make sense to train on the basis of a sport’s supposed attribute prototypes.

### Training Process. Pre-programmed or Co-adapted?

**Fig. 1:**
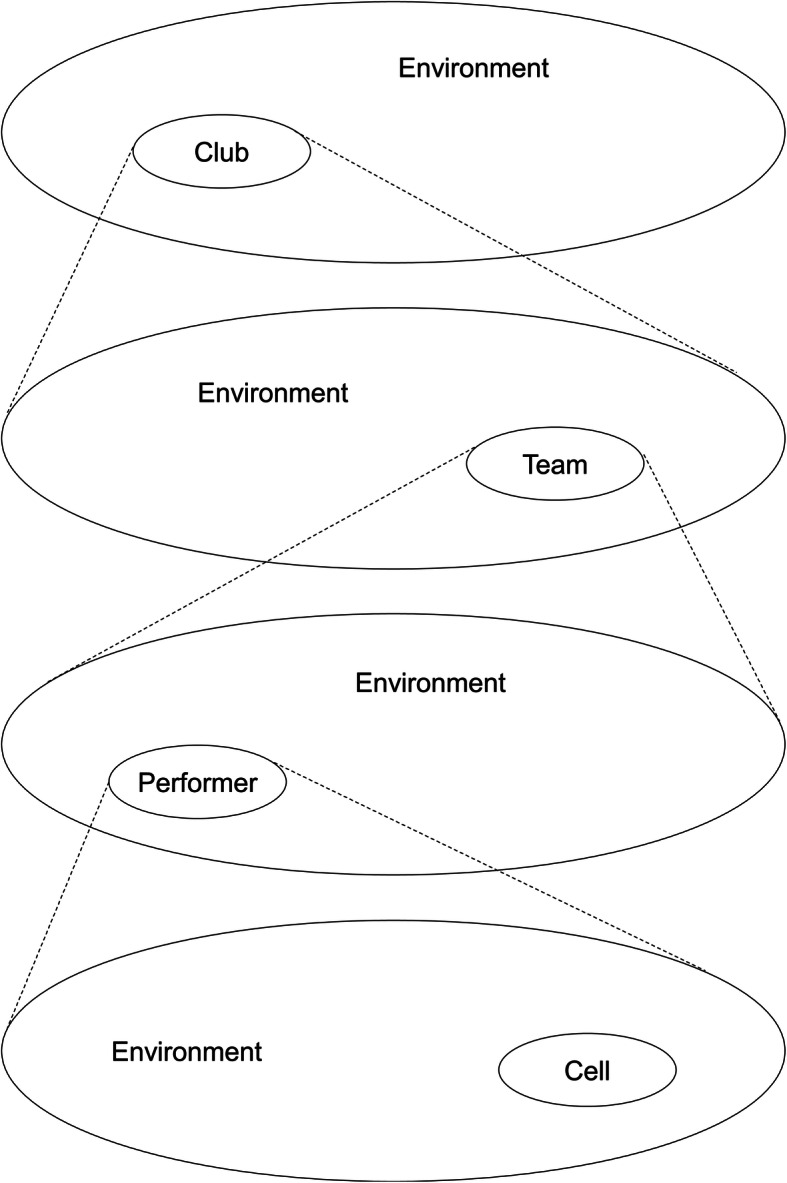
Nestedness of organization levels interacting at different timescales

It is assumed that individual and collective sport behavior emerges from the performer-environment interaction acting at different timescales [28] (Fig. 1). This means that when the environment changes, the behavior increases its probabilities to change as well, and when the environment keeps stable (e.g., similar opponents, similar constraints), the exploratory behavior ceases, and the emergence of new synergies too [24]. The diversification/complexification process is self-organized and cannot be pre-programmed. It requires not only varied challenging constraints but also novel technology to be evaluated [49]. Long-term training forecasts, as per conventional periodization models [2], are insufficiently responsive to flexibly adjust to such continuous and unpredictable co-adaptive performer-environment processes. In such contexts, it is the training process itself, and not the coach, that leads and shapes the coach-athlete interactions [50].

## Training or Synergizing? Contrast of Methodological Principles

### Training Programs or Methodological Criteria?

Fixed training programs assume the existence of decontextualized realities and ideal or prototypic static states in athletes/teams. As neither intrapersonal, interpersonal, or environmental constraints are repeated during training processes, the replacement of fixed training programs by methodological criteria based on complex dynamic principles seems advantageous [42]. The levels of fatigue, the emotional state, or the opponent’s behavior are only a few examples of constraints which demand continuous adjustments of training plans and which occur at shorter timescales than conventionally structured programs [2].

Complex dynamic principles (e.g., stability, instability, constraints, change of state…) are common to processes defined at multiple levels [42] and can be used as general methodological criteria. They are the fruit of compression, without fragmentation, of the huge complexity of levels (physiological, psychological, social, etc.) and timescales involved in sports training. Such criteria may embrace continuous, intertwined relationships between perception and action in different sport performance contexts.

### Skill Acquisition. Dominance of Instructional-Based or Environmental-Based Constraints?

While technological devices require instructions to change the task outcome (program, etc.), CAS orchestrate changes without instructions, i.e., as a result of the interaction with environmental constraints. Instructions are environmental information provided via social systems (e.g., coach) [28]. This information should be acknowledged, understood, and transformed into performer intentions in order to become a task constraint [24]. All task constraints, either informational or instructional, are then distributed between the performer and the environment, and thus, they are necessarily emergent, either by design (e.g., through instructions) or spontaneously, i.e., by self-organization. Thus, one cannot expect the same instruction to have the same effects on all performers. While some instructions provide direct information on how to perform the action (i.e., what to do or what to avoid), other types of environmental constraints (e.g., distance to the opponent, velocity of the ball) also constrain the performer’s affordances. While the former develops the dependency of the performer on instructions, the latter promotes the autonomy of the performer.

Instructions contribute to skill acquisition only if the CAS adequately understands the instruction and accurately transforms this instruction into personal intention.

In collective sports, both collective and individual performance is highly constrained by environmental factors [51–53]. The team's style, changing from match to match as a function of the opponents, match result, location (home or away), classification, etc., constrains the player’s individual performance (e.g., distance covered, amount of high-intensity runs, time of ball possession). Thus, individual performance, usually analyzed during competition, lacks relevance to the training of collective behavior. It is worth pointing out, here, that contrary to what is usually assumed, task constraints do not necessarily reduce, but may also increase, the degrees of freedom in a CAS [24]. Task constraints form boundaries around the exploration of certain action possibilities, while allowing the emergence of other exploration possibilities. When constraints reduce degrees of freedom, relevant information that coaches want performers to use is amplified.

### Teams or Players As Training Units in Collective Sports?

The fragmentation of the body into subsystems, which are trained separately (e.g., cardiovascular or neuromuscular exercise programs), and the division of performance into distinct attributes (e.g., strength, endurance, velocity, etc.), which are also trained separately, is a common practice in traditional training methodologies. Similarly, a key assumption in team sports is that collective performance is achieved through the sum of individual behaviors. In fact, soccer schools usually focus on training players, rather than training teams. Furthermore, performance evaluation is also predominantly player oriented [54].

In teams, conceptualized as superorganisms, performance emerges from the interaction among the individual parts [55]. The creation of team synergies requires the exposure of the whole set of players to challenging constraints. This promotes collective exploration, discovery, and stabilization of unique solutions based on intra-team interactions. In such training contexts, changing the set of constraints is the main driver of exploration behavior [56].

The emerging collective properties of teams cannot be assigned to any single player, in a similar way that life, as an emergent property of neurobiological systems, cannot be assigned to any specific subsystem of the organism (e.g., cardiovascular endocrine). In this sense, teams are not part of the context in which players perform innovatively and creatively but are the innovative and creative entity targeted by training designs [57]. That is, the target in team sports is the team, and the manipulation of constraints is addressed to increase the team’s potential diversity. This entails the development of networked team connectivity, the creation of new team synergies, and thus, the complexification of the team’s functionality. Burke et al. [58] define team adaptation as a change in team performance that leads to a functional outcome for the entire team and manifests through changed structures, capacities, behavior, and goal-directed actions of the whole team. This is distinct from different approaches in team sports, focused on the development of individual players’ diversity potential [56, 59].

Due to the nestedness of constraints, there is no need to reduce the training unit to individual players in team sports or to a subsystem in individual sports. Team collaborative properties like exploration [40, 41], degeneracy [60, 61], synergies [62], and synchronization [43, 63], developed through challenging and varied environments, adequately diversify individual behavior in a correlated way. While individual properties of players are important for building specific interactions within teams, these properties are best developed while playing in collective contexts. These collaborative properties may show up in a variety of game situations which can be defined at different levels and timescales, including player’s effectivities (speed, endurance, strength, etc.), player’s motivation, affection, flexibility, and creativity. The same rationale applied to collective sports can be applied to individual sports. Individual performers are also formed by collections of components and processes that interact within them, and with the environment, to satisfy a common purpose (survival in competition). Through challenging environmental contexts such components create new synergies promoting the development of their diversity potential, as has been shown in studies investigating the unintentional or spontaneous interpersonal synchronization of 100 m speed runners during competition [64]. The use of pacemakers, or rabbits, during long distance running is another example of how individual athletes increase their diversity potential in competition and beat their records.

### Role of the Coach. Prescribing Actions or Manipulating Constraints?

Assuming that sports are dynamic entities and that sport behavior is a product of the performer-environment system, which is irreproducible and highly unpredictable in competitive environments, the role of the coach, fixing task outcomes and prescribing actions, is under question. Coaches do not know all possible solutions of a task. In addition, prescriptions promote a power-dependency based coach-athlete relationship, a command-action based coupling, and a limited performer involvement.

As previously mentioned, instead of prescribing actions, coaches can manipulate constraints (personal or environmental) to promote the creativity (potential diversity/unpredictability) and autonomy of the performers. In opposition sports, as new intentions and tasks emerge continuously over very short time scales, due to the opponent’s behavior, performers act according to the newly perceived affordances and continuously shape new functional affordances. In this scenario, coaches’ prescriptions of actions might be counterproductive if competing with the performer’s perceived affordances. From another perspective, fixed prescriptions and programs may promote coach’s inattentional blindness to surrounding emergent information [65]. In such circumstances, coaches’ feedback focus is put on the results and not in the execution.

Athletes/teams and coach constitute a learning system, in which the coach is not only the manager of the training environment [50] but also a learning component. As long as training is focused on satisficing the diversity potential, training should be a co-adaptive process. The coach co-adapts, continuously adjusting the constraints to the athlete/team evolution. His or her work is mostly focused on selecting and designing the problems to be solved and providing adapted, varied, innovative, and sufficiently challenging tasks to develop the team complexification/diversification. Since actions emerge from the performer-environment system, athletes/teams must be co-designers of the training process rather than mere executers. In short, coaches constrain performers, and performers constrain coaches, which being challenged also enhance their diversity potential [66].

## Updating Underexplored Aspects by the CLA

The constraints-led approach (CLA), based on Newell’s model [67] and underpinned by the ecological dynamics theory and the principles of nonlinear pedagogy [68, 69], has been widely applied in motor learning and skill acquisition [17, 18, 25]. More recently, it has been also adapted to skill acquisition in achievement [70], opposition [71], and team sports [8] to enhance expertise and sport talent through representative training activities.

**Fig. 2:**
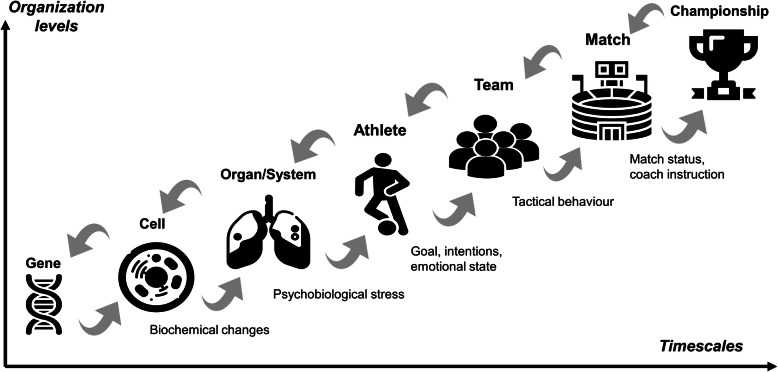
Levels of organization interacting through circular causality at different timescales

The CLA recommended an integrative approach of sport performance [72] and has been recently upgraded on the basis of two main characteristics of constraints: (1) they act at different nested timescales, and (2) they are circularly interdependent (bottom-up and top-down) [28, 73]. This means that organismic levels (genes, cells, tissues, organs, players, teams) are related through circular causality. In this way, it is emphasized to enlarge the skill acquisition and interpersonal coordination of the CLA, understood under the framework of the perception-action coupling (e.g., decision-making in technical and tactical behavior), to all training dimensions, including strength and conditioning, in a correlated way. Although previous authors have applied the CLA to strength and coordination [74], the property of interdependency, temporal nestedness, and circular causality of constraints acting at different timescales has yet to be implemented in integrative sport methodologies. These properties, interacting bottom-up and top-down, from social to biochemical and beyond, provide a basis to dilute the boundaries between traditional silos of sports training (e.g., technical, tactical, conditional, psychological) and to encourage the correlated development of capabilities. For instance, as illustrated in Fig. 2, a longer timescale coach instruction (high pressing), lasting minutes, during soccer matches produces a cascade of effects on shorter timescale processes; it drastically changes team tactics (defence high up the pitch), individual goals (one versus one), strategies (force mistakes to steal the ball), actions (high intensity), emotions (fears, anxieties), and physiological stress (high anaerobic activation). These top-down constrained processes are also related bottom-up. For instance, the psychobiological stress of a single player (e.g., center-back) may change his/her individual emotions (increased anxiety of being beaten by passes into the space behind), strategies (increasing the distance from the opponent), actions (stepping back to protect the goal), changing the whole team defence actions (withdraw and defend close to the own goal), and consequently, the coach instruction. The tactical anxiety of the center-back player will not be solved through individual strength and conditioning training. A coach with a good understanding of the complexity of the game and the properties of CAS may decide how to intervene effectively. Due to the interdependence of constraints, the psychobiological stress of one player is related to other constraints acting at different timescales. Interventions at long lasting constraints (e.g., team tactics) affect shorter lasting constraints (e.g., technical actions). For instance, instead of recommending strength and conditioning to reduce the physiological stress of the player, or recommend psychological training to reduce his/her fear, the coach may recommend further development of team defence synergies and/or stimulating the cover play of the goal keeper to compensate faster the overloading of individual players. This type of intervention further develops team synergies and may help the center-back to feel safer. Coaches’ competence is not simply based on knowing many recipes to respond to concrete problems but on understanding the principles that may help to decide and intervene effectively in each specific context. Complex problems cannot always be solved by simple solutions.

The idea of the nested organization of constraints is fundamental for integrative training methodologies. Independently of its origin (social, physiological, biochemical, psychological, biomechanical, etc.), constraints are related among them through timescales. This means that when the social (team) coordination is constrained, all other levels down (dyadic, interlimb, intermuscular, intramuscular, metabolic, etc.) are also constrained in a correlated way. In short, it is not a requirement to separately train endurance, strength capabilities, or motor skills in an isolated way. Through representative and contextually based tasks, such conditional capabilities and skills are already trained and developed in a correlated way. Although the previously mentioned levels and scales (social, psychological, physiological) are taken into account as environmental and personal constraints within CLA, their properties of interdependence, temporal nestedness, and circular causality have been neither hypothesized, nor elaborated in detail until now. Thus, it is not simply a problem of focusing on an enlarged spectrum of constraints but emphasizing the practical significance of their *interdependent properties*, *temporal nestedness*, *and circular causation*. This highly important ontology of constraints and its practical implications on connecting conditioning dimensions (development of strength, endurance, etc.) was, until now, not addressed within the framework of the CLA.

A different understanding of tasks and task constraints as they are conceived by the CLA has been also introduced. They are understood as systemic properties emerging from the organism-environment interaction that do not exist without performers’ intentions [28]. Due to the relation of performer’s intentions/goals, and the interdependence of such goals with longer-term personal constraints (personal values, motivation, fears) through circular causality [28], an effective selection of task constraints cannot ignore the motivation degree of performer’s intentions. Performer’s intentions are more stable when they are correlated with personal and social values, i.e., more stable constraints changing at longer timescales. Although CLA proposes affective learning designs [75], it does not refer to their circular interdependence with intentions and their mutual stabilizing role. The same task, performed simply with the intention of satisfying the coaches’ instructions, as opposed to a task performed with intrinsic motivation, may exert different effects on athletes/teams learning, conditioning, and creativity.

The increase of the diversity potential of athletes/teams, as a main training goal, is achieved through the individual/collective exploration and discovery of functional solutions through challenging constraints. But what it is meant by challenging constraints? In this context, challenging constraints is representative of sufficiently diverse/unpredictable environments capable of developing new synergies in teams/athletes. The degree of sufficiency can be defined and modified in situ for each task. Such new synergies promote new task constraints, and through circular causality, the continuous complexification of the performer-environment system leading to the dynamic conception of sport, as illustrated in Table 1. Such dynamic, highly individualized, and self-organizing processes cannot be pre-programmed, nor promoted, through repetitive contexts. As evident from recent publications, CLA proposes that task constraints should be representative of those experienced within a competitive performance environment, i.e., what are called *representative learning designs* [1, 62, 70, 76]*.* Thus, it seems crucial to emphasize the diversity of task constraints and not only their representativeness. In relation to the development of the sport discipline as a whole, this representativeness itself is a dynamical or changing property. Clear examples can be found in the evolution of game dynamics in team sports like soccer, basketball, or volleyball and the development of running strategies in track and field.

## Integrating Prevention and Performance Training

Sport methodologies usually distinguish between injury resilience and performance training. This is a relevant topic because, even in sports with extensive resources (e.g., soccer), previous authors have found evidence of ineffective practices [77]. Accordingly, despite focussed prevention training, injury rates are not necessarily reduced [78]. Previously, researchers interviewed 44 professional teams about their injury prevention strategies. Almost unanimously the interviewees rated eccentric exercise as the most effective modality to prevent injuries [13]. Yet, as already suggested, looking for a simple and easy answer to a complex problem remains a common mistake in sports training contexts [79]. The assumption, held by many practitioners, that doing, for example, three series of ten repetitions twice a week, represents a meaningful injury reduction strategy illustrates the simplistic, reductionist view of injury prevention.

Although a direct relation of injury prevention, or risk mitigation, with training methodologies is difficult to establish [75], some authors have found an association between injury rates and training and coaching styles [80]. These are some of the main benefits that complex approaches can bring to the safety and wellbeing of performers seeking to integrate prevention and performance training:

- Base training methodologies on updated scientific theoretical assumptions, not merely on experiential or pseudoscientific proposals. Performers are complex, nonlinear dynamic systems, and sports are dynamic entities. This improved understanding facilitates an updated and conceptually valid lens through which to devise effective targeting the comprehensive care of athletes.
- Methodological criteria should adapt to the intrinsic dynamics of performers and the environmental context, thereby avoiding the imposition of de-contextualized training programs that may increase stress and injury risk [81].
- The coupling between the performers' intrinsic dynamics and the proposed task dynamics enhances the coordination and efficacy of the learning process [45].
- Holistic workloads, avoiding fragmentation, may increase the efficiency of the training process and avoid overuse and overloading.
- Representative tasks improve coordination and perception-action coupling.
- The goal of complexification and diversification, in contrast with the maximization of attributes, avoids excessively monotonous repetitions and overuse.
- The principle of sufficing diversity avoids the application of excessive training workloads and prevents overtraining and injuries.
- The role of performers as training co-designers, not mere executers, may enhance injury prevention [82].
- Being challenged, coaches co-adapt [50, 83], increasing their diversity potential and resources.
- As there are no fixed performance attributes or prototypes and the diversity potential can be developed in many different ways (degeneracy property), overuse and overloading become unnecessary.
- Synergies can adapt, making compensatory reconfigurations at multiple levels, thereby avoiding premature fatigue and overloading [32, 83, 84].
- A variety of challenging constraints improves psycho-emotional factors (e.g., motivation, joy, well-being and adherence) and the health status of performers [85].
- The correlatedness and nestedness of constraints serve to improve training efficiency and enhance the recovery [28].
- The improvement of decision-making and the development of performer-environment couplings prevent contact injuries [86].

## Conclusion

Experiential and scientific knowledge, relating to sports training methodologies, has been historically influenced by reductionist models. Based on complex systems science and theories of biological evolution, we provide a systematization and update of theoretical and methodological principles to transform the understanding of the sports training process. This contribution is not another methodology; it simply seeks to promote the critical thinking of scientists, coaches, and practitioners to help them update or create safer and efficient interventions. Coaches and practitioners usually search for practical recipes, but the only recipe emerging from complex systems principles is that there are no fixed recipes. Functional methodologies and interventions in one context can be dysfunctional in another, and contexts are always unrepeatable and inevitably unique. Instead of focusing on practical recipes, the focus is put on understanding the systems (athletes/teams) properties and the principles that rule their interactions with the environment, keeping in mind the main aim of the process: developing the diversity/unpredictability potential of athletes/teams, that is, synergizing the system. As athletes/teams are conceived as CAS interacting nonlinearly with their environment, synergizing is best achieved through continuously modulating challenging and meaningful constraints. As task constraints emerge from personal (goals) and a subset of environmental constraints, athletes’/teams’ values and goals cannot be ignored. Exposure to challenging and meaningful contexts pushes the exploration and discovery of new synergies, promotes a co-adaptive process between coaches and performers, and transforms sports in dynamic entities.

The properties of interdependence, temporally nested organization, and circular causality of constraints can be used to satisfy integrative training purposes. By manipulating constraints at team level, a cascade of interdependent individual constraints acting at many levels (cognitive, emotional, systemic, organic, cellular, genetic) occurs in a correlated way. The intervention on slow changing constraints (e.g., values system) guarantees more stable effects than the intervention at faster changing constraints (e.g., motivation). Taking into account the circular causality among the temporarily nested constraints, it is possible to integrate not only bottom-up but also top-down at all levels of performance (including physiological and conditional). This integration, transferable to other fields, suggests a drastic break with the classical reductionism of sports training and presents fertile research opportunities for the future.

## Data Availability

Not applicable

## Footnotes

1 The process of learning the skills you need to do a particular job or activity

2 To combine or work together in order to be more effective

3 In the CLA, constraints refer to boundary conditions or limitations that promote the emergence of synergies restricting the degrees of freedom of teams/athletes [24].

4 By “fittest” Darwin meant “better adapted for the immediate, local environment.”

5 Please notice that we avoid here to mention the classical performance attributes or dimensions (e.g., strength, endurance, velocity), sub-dimensions (explosive strength, reaction speed, etc.), or combinations of them (agility, strength endurance, etc.).

## References

[1] Renshaw I, Davids K, Newcombe D, Roberts W (2019). The constraints-led approach: principles for sports coaching and practice design.

[2] Kiely J. Periodization theory: confronting an inconvenient truth. Sport Med - Open [Internet]. 2018;48:753–764. Available from: 10.1007/s40279-017-0823-y. 10.1007/s40279-017-0823-y PMC5856877 29189930

[3] Stoszkowski J, Collins D (2015). Sources, topics and use of knowledge by coaches. J Sport Sci..

[4] Fullagar HHK, Mccall A, Impellizzeri FM, Favero T, Coutts AJ. The translation of sport science research to the field: a current opinion and overview on the perceptions of practitioners, researchers and coaches. Sport Med [Internet]. 2019; Available from: 10.1007/s40279-019-01139-0. 10.1007/s40279-019-01139-0 31214978

[5] Bailey RP, Madigan DJ, Cope E, Nicholls AR. The prevalence of pseudoscientific ideas and neuromyths among sports coaches. Front Psychol [Internet]. 2018;9, 641 Available from: https://www.frontiersin.org/article/10.3389/fpsyg.2018.00641. 10.3389/fpsyg.2018.00641 PMC5941987 29770115

[6] Balagué N, Pol R, Guerrero I. Science or pseudoscience of physical activity and sport? Apunt Educ Física i Esports. 2019:129–36.

[7] Bradley P, Ade J (2018). Are current physical match performance metrics in elite soccer fit for purpose or is the adoption of an integrated approach needed?. Int J Sports Physiol Perform..

[8] Correia V, Carvalho J, Araújo D, Pereira E, Davids K. Principles of nonlinear pedagogy in sport practice. Phys Educ Sport Pedagog [Internet]. 2019;24:117–32. Available from: 10.1080/17408989.2018.1552673.

[9] Butterfield T (2010). Eccentric exercise in vivo: strain-induced muscle damage and adaptation in a stable system. Exerc Sport Sci Rev..

[10] McCall A, Carling C, Davison M, Nedelec M, Le Gall F, Berthoin S (2015). Injury risk factors, screening tests and preventative strategies: a systematic review of the evidence that underpins the perceptions and practices of 44 football (soccer) teams from various premier leagues. Br J Sports Med [Internet]..

[11] Hibbert O, Grant A, Beers A (2008). A systematic review of the effectiveness of eccentric strength training in the prevention of hamstring muscle strains in otherwise healthy individuals. North Am J Sport Phys Ther..

[12] Goode AP, Reiman MP, Harris L, Delisa L, Kauffman A, Beltramo D (2015). Eccentric training for prevention of hamstring injuries may depend on intervention compliance : a systematic review and meta-analysis. Br J Sports Med..

[13] McCall A, Carling C, Nedelec M, Davison M, Le Gall F, Berthoin S (2014). Risk factors, testing and preventative strategies for non-contact injuries in professional football: current perceptions and practices of 44 teams from various premier leagues. Br J Sports Med..

[14] Mujica I (2017). Quantification of training and competition loads in endurance sports: methods and applications. Int J Sports Physiol Perform..

[15] Borresen J, Lambert MI (2009). The quantification of training load, the training response and the effect on performance. Sport Med..

[16] Berthelot G, Sedeaud A, Marck A, Marc A (2015). Has Athletic Performance Reached its Peak?. Sport Med..

[17] Davids K, Button C, Bennett SJ (2008). Dynamics of skill acquisition: a constraints-led approach.

[18] Renshaw I, Davids K, Savelsbergh GJP (2010). Motor learning in practice: a constraints-led approach.

[19] Pross A. What is life?: How chemistry becomes biology. Oxford: Oxford University Press; 2016.

[20] Bar-Yam Y (2003). Complex systems insights to building effective teams. Int J Comput Sci Sport..

[21] Davids K, Araújo D, Seifert L, Orth D. Expert performance in sport: an ecological dynamics perspective. Routledge Handb Sport Expert. London: Routledge; 2015. p. 156–70.

[22] Passos P, Davids K, Araújo D, Paz N, Minguéns J, Mendes J. Networks as a novel tool for studying team ball sports as complex social systems. J Sci Med Sport [Internet]. 2011;14:170–6. Available from: 10.1016/j.jsams.2010.10.459. 10.1016/j.jsams.2010.10.459 21145787

[23] McLean S, Salmon PM, Gorman AD, Read GJM, Solomon C (2017). What’s in a game? A systems approach to enhancing performance analysis in football. PLoS One..

[24] Torrents C, Balagué N, Ric A, Hristovski R. The motor creativity paradox: constraining to release degrees of freedom. Psychol Aesthet Creat Arts. 2020. 10.1037/aca0000291.

[25] Handford C, Davids K, Bennett S, Button C (1997). Skill acquisition in sport: some applications of an evolving practice ecology. J Sports Sci..

[26] Searle J, Göranzon B, Florin M (1990). Cognitive science and the computer metaphor. Artifical Intell Cult Lang Educ Work.

[27] Araújo D, Davids K. Team synergies in sport: theory and measures. Front Psychol [Internet]. 2016;7:1449. Available from: https://www.frontiersin.org/article/10.3389/fpsyg.2016.01449. 10.3389/fpsyg.2016.01449 PMC5030782 27708609

[28] Balagué N, Pol R, Torrents C, Ric A, Hristovski R. On the relatedness and nestedness of constraints. Sport Med - Open [Internet]. 2019;5:6. Available from: 10.1186/s40798-019-0178-z. 10.1186/s40798-019-0178-z PMC6370894 30742241

[29] Riley M, Richardson M, Shockley K, Ramenzoni V. Interpersonal synergies. Front Psychol [Internet]. 2011;2:38. Available from: https://www.frontiersin.org/article/10.3389/fpsyg.2011.00038. 10.3389/fpsyg.2011.00038 PMC3110940 21716606

[30] Kelso JAS. Principles of coordination: synergies of synergies! Complex Syst Sport Int Congr Link Theory Pract. 2017. p. 13.

[31] Latash M. Human movements: synergies, stability, and agility. Biomech Anthr Syst. Cham: Springer; 2019. p. 135–54.

[32] Balagué N, González J, Javierre C, Hristovski R, Aragonés D, Álamo J (2016). Cardiorespiratory coordination after training and detraining. A principal component analysis approach. Front Physiol..

[33] Balagué N, Hristovski R, Garcia S, Aragonés D, Razon S, Tenenbaum G (2015). Intentional thought dynamics during exercise performed until volitional exhaustion. J Sports Sci..

[34] Kelso JAS. Synergies: atoms of brain and behavior. Prog Mot Control. Boston: Springer; 2009. p. 83–91. 10.1007/978-0-387-77064-2_5 19227496

[35] Haken H, Yates FE (1987). Synergetics: an approach to self-organization. Self-organizing Syst Emerg order.

[36] Edelman GM, Gally JA (2001). Degeneracy and complexity in biological systems. Proc Natl Acad Sci..

[37] Bovier A, Den Hollander F (2016). Metastability: a potential-theoretic approach.

[38] Hristovski R. Unpredictability in competitive environments. In: Torrents C, Passos P, Cos F, editors. Complex Syst Sport Int Congr Link Theory Pract. Frontiers; 2017. p. 9–12.

[39] Ramos A, Coutinho P, Silva P, Davids K, Mesquita I (2017). How players exploit variability and regularity of game actions in female volleyball teams. Eur J Sport Sci..

[40] Ric A, Torrents C, Gonçalves B, Sampaio J, Hristovski R (2016). Soft-assembled multilevel dynamics of tactical behaviors in soccer. Front Psychol..

[41] Torrents C, Ric A, Hristovski R, Torres-Ronda L, Vicente E, Sampaio J (2016). Emergence of exploratory, technical and tactical behavior in small-sided soccer games when manipulating the number of teammates and opponents. PLoS One..

[42] Hristovski R, Balagué N, Schöllhorn W, Davids K, Hristovski R, Araújo D, Balagué N, Button C, Passos P (2014). Basic notions in the science of complex systems and nonlinear dynamics. Complex Syst Sport.

[43] Duarte R, Araújo D, Correia V, Davids K, Marques P, Richardson MJ (2013). Competing together: assessing the dynamics of team–team and player–team synchrony in professional association football. Hum Mov Sci [Internet]..

[44] Silva P, Garganta J, Araújo D, Davids K, Aguiar P (2013). Shared knowledge or shared affordances? Insights from an ecological dynamics approach to team coordination in sports. Sport Med..

[45] Kelso JAS (1995). Dynamic patterns: The self-organization of brain and behavior.

[46] Shannon C, Weaver W (1949). The mathematical theory of communication.

[47] Ashby WR (1956). An introduction to cybernetices.

[48] Simon HA (1956). Rational choice and the structure of the environment. Psychol Rev..

[49] Silva P, Duarte R, Esteves P, Travassos B, Vilar L. Application of entropy measures to analysis of performance in team sports. Int J Perform Anal Sport [Internet]. 2016;16:753–68. Available from: 10.1080/24748668.2016.11868921.

[50] Orth D, van der Kamp J, Button C. Learning to be adaptive as a distributed process across the coach–athlete system: situating the coach in the constraints-led approach. Phys Educ Sport Pedagog [Internet]. 2019;24:146–61. Available from: 10.1080/17408989.2018.1557132.

[51] Liu H, Gómez M, Gonçalves B, Sampaio J. Technical performance and match-to-match variation in elite football teams. 2016;34:509–518. 10.1080/02640414.2015.1117121 26613399

[52] Lago C (2009). The influence of match location, quality of opposition, and match status on possession strategies in professional association football. J Sport Sci..

[53] Gregson W, Drust B, Atkinson G, Salvo VD (2010). Match-to-match variability of high-speed activities in premier league soccer. Int J Sports Med..

[54] Haugen T, Seiler S (2015). Physical and physiological testing of soccer players: why, what and how should we measure?. Sportscience..

[55] Duarte R, Araújo D, Correia V, Davids K (2012). Sports teams as superorganisms. Sport Med. Springer.

[56] Ric A, Hristovski R, Gonçalves B, Torres L, Sampaio J, Torrents C. Timescales for exploratory tactical behaviour in football small-sided games. J Sports Sci [Internet]. 2016;34:1723–30. Available from: 10.1080/02640414.2015.1136068. 10.1080/02640414.2015.1136068 26758958

[57] Reiter-Palmon R (2017). Team creativity and innovation.

[58] Burke CS, Stagl KC, Salas E, Pierce L, Kendall D (2006). Understanding team adaptation: a conceptual analysis and model. J Appl Psychol..

[59] Ric A, Torrents C, Gonçalves B, Torres-Ronda L, Sampaio J, Hristovski R. Dynamics of tactical behaviour in association football when manipulating players’ space of interaction. PLoS One. 2017;12(7):e0180773. 10.1371/journal.pone.0180773 PMC5510826 28708868

[60] Seifert L, Wattebled L, Herault R, Poizat G, Adé D, Gal-Petitfaux N (2014). Neurobiological degeneracy and affordance perception support functional intra-individual variability of inter-limb coordination during ice climbing. PLoS One..

[61] Pinder R, Davids K, Renshaw I, Araújo D (2011). Representative learning design and functionality of research and practice in sport. J Sport Exerc Psychol..

[62] Passos P, Milho J, Button C (2018). Quantifying synergies in two-versus-one situations in team sports: an example from Rugby Union. Behav Res Methods. Behavior Research Methods.

[63] López-Felip MA, Davis TJ, Frank TD, Dixon JA (2018). A cluster phase analysis for collective behavior in team sports. Hum Mov Sci..

[64] Varlet M, Richardson MJ. What would be Usain Bolt’s 100-meter sprint world record without Tyson Gay? Unintentional interpersonal synchronization between the two sprinters. J Exp Psychol Hum Percept Perform. 2015;41(1):36–41. 10.1037/a0038640 25559749

[65] Memmert D, Furley P (2007). “I Spy with My Little Eye!”: breadth of attention, inattentional blindness, and tactical decision making in team sports. J Sport Exerc Psychol..

[66] Hristovski R. A constraints-based intervention in boxing. In: I. Renshaw, K. Davids GS, editor. Mot Learn Pract a constraints-led approach. London: Roudledge; 2010. p. 211–20.

[67] Newell KM (1986). Constraints on the development of coordination. Mot Dev Child Asp Coord Control..

[68] Araujo D, Davids K, Hristovski R (2006). The ecological dynamics of decision making in sport. Psychol Sport Exerc..

[69] Chow Y, Davids K, Hristovski R, Araújo D, Passos P (2011). Nonlinear pedagogy: learning design for self-organizing neurobiological systems. New Ideas Psychol..

[70] Seifert L, Orth D, Button C, Brymer E, Davids K. An ecological dynamics framework for the acquisition of perceptual–motor skills in climbing. Extrem Sport Med. Cham: Springer; 2017. p. 365–82.

[71] Carvalho J, Araújo D, Travassos B, Esteves P, Pessanha L, Pereira F, et al. Dynamics of players’ relative positioning during baseline rallies in tennis. J Sports Sci [Internet]. 2013;31:1596–605. Available from: 10.1080/02640414.2013.792944. 10.1080/02640414.2013.792944 23687954

[72] Glazier P (2017). Towards a grand unified theory of sports performance. Hum Mov Sci..

[73] Hristovski R, Aceski A, Balague N, Seifert L, Tufekcievski A, Cecilia A. Structure and dynamics of European sports science textual contents: analysis of ECSS abstracts (1996–2014). Eur J Sport Sci. 2016;17(1):19–29. 10.1080/17461391.2016.1207709 27460778

[74] Bosch F, Cook K (2015). Strength training and coordination: an integrative approach.

[75] Headrick J, Renshaw I, Davids K, Pinder RA, Araújo D (2015). The dynamics of expertise acquisition in sport: the role of affective learning design. Psychol Sport Exerc..

[76] Woods CT, McKeown I, Shuttleworth RJ, Davids K, Robertson S (2019). Training programme designs in professional team sport: an ecological dynamics exemplar. Hum Mov Sci..

[77] Fanchini M, Steendahl IB, Impellizzeri FM., Pruna R, Dupont G, Coutts AJ, McCall A. Exercise-based strategies to prevent muscle injury in elite footballers: a systematic review and best evidence synthesis. Sport Med [Internet]. 2020; Available from: 10.1007/s40279-020-01282-z. 10.1007/s40279-020-01282-z 32185630

[78] Ekstrand J, Waldén M, Hägglund M. Hamstring injuries have increased by 4% annually in men’s professional football, since 2001: a 13-year longitudinal analysis of the UEFA Elite Club injury study. Br J Sports Med [Internet]. 2016;50:731–7. Available from: http://www.ncbi.nlm.nih.gov/pubmed/26746908. 10.1136/bjsports-2015-095359 26746908

[79] Sturmberg J, Topolski S (2014). For every complex problem, there is an answer that is clear, simple and wrong: and other aphorisms about medical statistical fallacies. J Eval Clin Pract..

[80] Ekstrand J, Lundqvist D, Lagerbäck L, Vouillamoz M, Papadimitiou N, Karlsson J. Is there a correlation between coaches’ leadership styles and injuries in elite football teams? A study of 36 elite teams in 17 countries. Br J Sports Med. 2018;52(8):527–31. 10.1136/bjsports-2017-098001 PMC5890645 29056596

[81] Ekstrand J, Gillquist J, Möller M, Oberg B, Liljedahl S-O (1983). Incidence of soccer injuries and their relation to training and team success. Am J Sports Med..

[82] Pol R, Hristovski R, Medina D, Balague N. From microscopic to macroscopic sports injuries. Applying the complex dynamic systems approach to sports medicine: a narrative review. Br J Sports Med. 2018;0:1–8. 10.1136/bjsports-2016-097395 29674346

[83] Hristovski R, Venskaityte E, Vainoras A, Balagué N, Vazquez P (2010). Constraints-controlled metastable dynamics of exercise-induced psychobiological adaptation. Medicina (B Aires).

[84] Vázquez P, Hristovski R, Balagué N (2016). The path to exhaustion: time-variability properties of coordinative variables during continuous exercise. Front Physiol..

[85] Hutto DD, Kirchhoff MD, Renshaw I, Capuccio M (2019). Emotions on the playing field. Handb Embodied Cogn Sport Psychol.

[86] Leventer L, Dicks M, Duarte R, Davids K, Araújo D (2015). Emergence of contact injuries in invasion team sports: an ecological dynamics rationale. Sport Med..

